# Empathy-Driven Humanization: Employment Instability, Burnout, and Work Engagement Among Temporary Nurses in a Sustainable Workforce Model

**DOI:** 10.3390/nursrep15070223

**Published:** 2025-06-20

**Authors:** Sonia Prieto-de Benito, Carlos Ruíz-Núñez, Juan Pablo Hervás-Pérez, Cayetana Ruíz-Zaldibar, Fidel López-Espuela, Raquel Caballero de la Calle, Ivan Herrera-Peco

**Affiliations:** 1 Hospital Clínico Universitrio de Valladolid, Av. Ramón y Cajal, 3, 47003 Valladolid, Spain; sprietodb104@gmail.com; 2 Centro de Emergencias Sanitarias 061, Unidad de Innovación, 29590 Málaga, Spain; 3 Faculty of Health Sciences-HM Hospitals, University Camilo José Cela, Urb. Villafranca del Castillo, 49, Villanueva de la Cañada, 28692 Madrid, Spain; jphervas@ucjc.edu (J.P.H.-P.); crzaldibar@ucjc.edu (C.R.-Z.); 4 Instituto de Investigación Sanitaria HM Hospitales, 28015 Madrid, Spain; 5 Metabolic Bone Diseases Research Group, Nursing Department, Nursing and Occupational Therapy College, University of Extremadura, 10003 Cceres, Spain; fidellopez@unex.es; 6 Nurse-In Research Group, Faculty of Health Sciences, Universidad Alfonso X El Sabio, Villanueva de la Cañada, 28691 Madrid, Spain; rdelacab@uax.es; 7 Socialhealthcare-UAX Research Group, Faculty of Health Sciences, Universidad Alfonso X El Sabio, Villanueva de la Cañada, 28691 Madrid, Spain

**Keywords:** burnout, empathy, humanization of care, engagement, nurses, employment instability

## Abstract

**Background/Objectives:** Employment instability is increasingly recognized as an organizational stressor, yet its combined effect on nurse burnout, humanized care, and work engagement is poorly quantified. This study investigates those relationships and tests a serial mediation model linking contract instability, burnout, humanization, and engagement in Spanish hospital nurses. **Methods:** A nationwide cross-sectional survey was completed by 400 fixed-term nurses between March and May 2025. The data included demographics, number of contracts signed during 2024, and scores on the Maslach Burnout Inventory (MBI), Utrecht Work Engagement Scale (UWES), and Health Professionals’ Humanization Scale (HUMAS). Spearman coefficients described the bivariate relations. **Results:** Burnout correlated positively with both contract count (r = 0.42, *p* = 0.039) and years of experience (r = 0.74, *p* = 0.040). Work engagement was inversely associated with instability (r = –0.62, *p* = 0.018). Humanized care was strongly and negatively related to burnout (r = –0.61, *p* = 0.032), particularly in sociability and self-efficacy dimensions. **Discussion:** Contractual precarity elevates burnout, erodes perceptions of humanized care, and, through this erosion, suppresses nurse engagement. Stabilizing workforce arrangements and strengthening empathy-centered skills may mitigate these effects and foster a socially sustainable nursing workforce.

## 1. Introduction

The number of nursing professionals in Spain has risen steadily over the last decade. According to the National Statistics Institute, the workforce grew from 306,611 registered nurses in 2018 to 336,321 in 2022, representing an average net increase of 6000–7000 graduates per year [1]. During the same period, demand on the healthcare system intensified: annual admissions now exceed four million, with 82 million outpatient visits, 3.4 million surgeries, and 21 million emergency encounters [2].

Despite this growth, nurse-to-patient ratios remain below recommended levels. Whereas evidence suggests an optimal ceiling of 6–8 patients per nurse, Spanish wards commonly assign 10–20 patients, depending on the facility and shift [3]. These staffing shortfalls have heightened concerns about care quality, avoidable mortality, and clinical error.

These pressures intensify the nursing role and contribute to increasingly stressful work environments. Employment instability in nursing is best captured as the cumulative number of fixed-term (temporary) contracts a nurse signs within a given period. Each additional contract represents a new episode of uncertainty, magnifying psychological strain and eroding continuity of care. High workloads and constant tension are well-recognized precursors of emotional exhaustion and long-term burnout [4,5]. This state of exhaustion, when prolonged, may result in a gradual decline in professional skills and coping abilities [6].

Several factors influence burnout among healthcare professionals, spanning sociodemographic, occupational, personal, and interpersonal domains [7,8]. When clinicians cannot marshal adequate resources to meet escalating demands, maladaptive coping responses surface, fostering absenteeism, turnover, and eroding the team climate [9,10]. Burnout also imposes direct financial costs on organizations: affected nurses are more prone to performance lapses that jeopardize patient safety [3] and may ultimately exit the profession to protect their health [11]. Budget-driven staffing cuts, heavier caseloads, and early retirement feed this cycle, amplifying burnout and suppressing work engagement.

Empathy, as a core component of humanized care, plays a pivotal role in shaping the emotional connection between healthcare professionals and their patients. Defined as the ability to understand and share the emotional states of others, empathy fosters more meaningful interactions and improves clinical outcomes [12,13].

Work engagement is likewise protective. Conceptualized as a positive, work-related state of vigor, dedication, and absorption, engagement correlates with better employee well-being and higher patient satisfaction [14]. Among temporary nurses navigating unstable environments, empathy appears to sustain engagement by fostering intrinsic motivation and prosocial behavior [15,16]. Numerous studies have shown that work engagement not only influences employee well-being, but also has a positive impact on patient satisfaction [17]. Engaged healthcare professionals tend to develop essential competencies such as physical and psychological involvement, empathy, communication skills, and the ability to provide humanized care [18].

Among the personal resources that contribute to work engagement, empathy has emerged as a significant psychological asset. It not only enhances interpersonal relationships but also strengthens emotional resilience in the face of occupational stress. In temporary nursing staff, who often navigate rapidly changing environments and shifting responsibilities, the ability to empathize with patients and colleagues may help preserve a sense of dedication and purpose [15]. Research suggests that empathetic individuals are more likely to maintain engagement even under pressure, as they find intrinsic motivation through meaningful connections and prosocial behavior [16,19]. As such, empathy may act as a moderator in the relationship between burnout and engagement.

Humanized care is a mutual process of communication and emotional support aimed at understanding and nurturing the essential spirit of life [18]. It is a multifactorial construct involving both cognitive and affective elements, whereby the nurse adopts a holistic and integrative approach to care, placing the patient at the center of the healthcare system [17,18]. Empathy reduces emotional exhaustion and improves overall well-being among healthcare professionals [18].

However, maintaining such engagement has become increasingly challenging in recent years due to the rise in temporary employment contracts among healthcare staff. This increase has led to high mobility among nurses, with many not remaining long enough in a single department to establish effective working relationships. A study conducted in the United Kingdom found that the number of nurses working under temporary contracts has increased significantly in recent years [20], directly impacting the continuity and quality of care provided to patients [21,22].

Job insecurity, salary erosion, service rotations, and mounting case complexity further degrade nurses’ quality of life [23]. Missed nursing care—errors of omission tied to temporary staffing—raises the incidence of falls and medication errors and fosters professional insecurity [20,24]. Chronic understaffing also fuels “dehumanization,” wherein patients are treated mechanistically, and nurses experience moral disengagement [18]. Irregular shift work compounds these risks, disrupting circadian rhythms and heightening cardio-metabolic and psychological morbidity [25,26]. Effective coping strategies and autonomy can temper these effects [27].

Job Demands–Resources (JD–R) theory offers a coherent lens through which the present variables can be interpreted. JD–R theory posits a health-impairment process, where excessive job demands such as workload, irregular shifts, and contractual uncertainty exhaust workers, and a motivational process whereby job and personal resources energize engagement [28]. Empirical evidence in hospital settings shows that relational resources like empathy, optimism, and self-efficacy attenuate the impairment path and redirect energy toward prosocial outcomes [29,30]. Recent work with Spanish nurses further demonstrates that resilience and engagement serially mediate the link between emotional demands and burnout [31]. We therefore conceptualize empathy and the broader construct of humanization as personal resources that may offset situational stressors and sustain engagement, an assumption that underpins the serial-mediation model tested in the present study.

Guided by the JD-R framework, the present study pursued the main goal of analyzing the impact of employment instability on burnout, work engagement, and the perception of humanized, empathic care among Spanish hospital nurses, and to propose a serial mediation model linking these variables. The specific aims were as follows: [i] a higher **count of temporary contracts** will predict greater burnout, which in turn will relate to lower humanization of care and reduced work engagement; [ii] to examine the association between burnout and work engagement; [iii] to assess whether burnout relates to nurses’ perceptions of humanized, empathic care; and [iv] to explore a serial mediation model in which employment instability increases burnout, which then diminishes humanization and, consequently, engagement.

Despite extensive evidence on workload and burnout in nursing, no prior study has specifically examined the compounded impact of temporary contracts, irregular rotations, and disorganized shifts on burnout, humanized care, and engagement in Spanish nurses. Addressing this gap will inform staffing policies and targeted interventions to safeguard both professional well-being and patient safety.

## 2. Materials and Methods

### 2.1. Study Design

We carried out an observational, descriptive, cross-sectional study exclusively targeting hospital nurses employed on fixed-term contracts in Spain. The aim to focus exclusively on fixed-term nurses stemmed from the study aim: to isolate the psychosocial impact of employment instability without the confounding influence of mixed contractual conditions. Concentrating on a single contract category enhances internal validity and yields clearer estimates of the instability–burnout–engagement pathway.

### 2.2. Participants

Data were gathered from public and private acute-care hospitals across Spain. To be eligible, participants had to [a] be registered nurses, [b] hold an active fixed-term contract of at least three months’ duration, and [c] be working directly with patients at the time of the survey. Nurses with permanent, interim, per-diem, agency, or self-employed contracts were excluded.

The sample was calculated with G*Power 3.1.9.7 [Heinrich-Heine-Universität Düsseldorf], where we specified an effect size r = 0.15, α = 0.05, and power of 0.80. We anticipate up to 150% attrition or unusable responses, and finally we obtained a sample of 384 participants.

### 2.3. Data Collection and Instruments

Data were collected through an electronic questionnaire was programmed with Microsoft Forms [Microsoft Corporation, Redmond, WA, USA], via a web link. The form was open from 1 March to 31 May 2025, by using an online platform that was accessible from any device with an internet connection, such as smartphones, computers, or tablets. To reduce the risk of self-selection bias inherent in web-based surveys, we [a] shared the invitation through multiple independent channels—professional colleges and social-media groups—to reach nurses with diverse technological access; [b] the invitation was redacted in a neutral tone to avoid attracting only highly dissatisfied or highly engaged staff; and [c] limited submissions to one response.

The instrument selected to collect data was structured online questionnaire composed of two sections; one regarding the sociodemographic data and the second one related to the main variables of the study measured through questionnaires.

Sociodemographic information was obtained related to gender, age, years of professional experience, fixed-term contracts they had signed between 1 January and 31 December 2024 (range 0–12), type of hospital (public or private), and work shift modality using an ad hoc questionnaire.

The second section contained psychometric instruments, including three validated scales. Maslach Burnout Inventory [MBI] [28]: This tool measures the symptoms associated with Burnout Syndrome. It comprises three dimensions: emotional exhaustion [α = 0.88], depersonalization [α = 0.69], and reduced personal accomplishment [α = 0.84] [29]. Emotional exhaustion is assessed using 9 items. Depersonalization is evaluated with 5 items. Reduced personal accomplishment is assessed with 8 items.

Utrecht Work Engagement Scale [UWES] [14], this scale assesses the work engagement and consists of three dimensions: Vigor [6 items], dedication [5 items], and absorption [6 items]. Cronbach’s alpha reliability indexes are as follows: vigor [α = 0.82], dedication [α = 0.86], and absorption [α = 0.8]

Health Professionals’ Humanization Scale [HUMAS] [15] was used to assess the humanization in healthcare practice through five dimensions: optimism [positive outlook] [ω = 0.86], sociability [empathic and assertive relationships] [ω = 0.86], emotional understanding [ω = 0.88], self-efficacy [confidence in one’s abilities] [ω = 0.86], and affection toward others [ω = 0.89]. McDonald’s omega was calculated to estimate the reliability of each of the subscales, and the complete scale was [ω = 0.88].

### 2.4. Ethical Considerations

The study adhered to the Declaration of Helsinki and received approval from the Research Ethics Committee of Universidad Alfonso X el Sabio [Ref. 2022_2/129]. Participation was anonymous and voluntary; respondents provided electronic informed consent and could withdraw at any stage without consequence. Confidentiality was protected through encrypted data storage and restricted access.

### 2.5. Data Analysis

Statistical analysis were conducted with IBM SPSS Statistics 23 (IBM Corp., Armonk, NY, USA). We first computed descriptive statistics (mean, median, standard deviation, and 95% confidence interval) for quantitative variables and absolute and relative frequencies for qualitative variables. Kolmogorov–Smirnov test indicated departures from normality for several key variables (*p* < 0.05), and subsequent bivariate relationships were examined with Spearman’s rank-order correlations (ρ). Differences between two independent groups (e.g., public vs. private hospital) were assessed with the Mann–Whitney U test, while associations among categorical variables were explored with χ^2^ tests and contingency coefficients.

The hypothesized serial mediation (employment instability → burnout → humanization → engagement) was evaluated with Hayes’s PROCESS macro v 4.2 (Model 6). Employment instability entered the model as a continuous count variable; no categorical predictors were included in any equation. All paths were estimated via ordinary-least-squares regression with 5000 bias-corrected bootstrap samples to obtain robust 95% confidence intervals for indirect effects. Standardized (β) and unstandardized (B) coefficients, standard errors, and bootstrap confidence intervals are reported.

To test the robustness of the model against potential non-linearity, we performed a sensitivity analysis in which the contract-count variable was recoded into three ordinal terciles (0–1, 2–3, and ≥4). The mediation was re-estimated with the same bootstrap procedure; full results appear in Appendix A.3. Statistical significance was set at *p* < 0.05 for all tests.

## 3. Results

### 3.1. Sample Description

A total of 453 responses were received. After applying the contract-type criterion and removing incomplete questionnaires, 400 fully completed surveys from temporary nurses were retained for analysis [88.3%]. As shown in Table 1, a total of 400 nursing professionals participated in the study, of whom 292 were women (73%) and 108 were men (27%).

Regarding the type of hospital, 58% of the nurses reported working in public hospitals, while 42% were employed in private institutions. Most respondents worked rotating shifts [65.3%, n = 261]; 31.8% (n = 127) were on permanent day shifts, and 3.0% (n = 12) were on permanent afternoon shifts.

In relation to the descriptive statistics for each dimension of work engagement and humanization of care, within the UWES subscales, vigor obtained the highest mean score (M = 3.13; SD = 0.42; 95% CI = 3.09–3.17), followed closely by absorption (M = 2.97; SD = 0.49) and dedication (M = 2.95; SD = 0.51) (Table 2). These values reflect a generally moderate level of engagement among participants, with a tendency toward sustained energy and involvement in their professional activity.

Regarding the humanization scale, the highest-scoring subdimension was optimism (M = 2.67; SD = 0.42; 95% CI = 2.63–2.71), followed by sociability (M = 2.61; SD = 0.37), self-efficacy (M = 2.51; SD = 0.33), and emotional understanding (M = 2.46; SD = 0.36). The lowest average score was observed for affection (M = 2.33; SD = 0.41), suggesting that although humanization is generally present, the affective dimension may be less developed or more vulnerable under working conditions such as those associated with temporary employment or a high workload (Table 2).

### 3.2. Correlational Analysis Between Study Variables

The associations between sociodemographic and occupational characteristics and the main outcome variables, burnout, engagement, and humanization, were analyzed using Spearman’s correlation for continuous variables and chi-square tests with contingency coefficients for categorical ones.

Among the continuous variables, years of professional experience showed a strong and statistically significant positive correlation with burnout (r = 0.74; *p* = 0.04), indicating that more years in the profession may increase emotional exhaustion. The number of temporary contracts signed in the past year was also positively associated with burnout (r = 0.42; *p* = 0.039) and negatively with overall engagement (r = −0.62; *p* = 0.018), especially affecting the vigor (r = −0.49; *p* = 0.025), dedication (r = −0.41; *p* = 0.033), and absorption (r = −0.39; *p* = 0.041) subdimensions. No relevant associations were observed between age and the main outcomes (Table 3).

### 3.3. Effects of Burnout on Work Engagement and Humanization of Care

We observed an inverse correlation between emotional exhaustion and global work engagement (r = −0.60; *p* = 0.015) and the self-efficacy component of humanization (r = −0.66; *p* < 0.001). At the construct level, total burnout also predicted lower engagement (r = −0.54; *p* = 0.028) and lower overall humanization (r = −0.61; *p* = 0.032). By contrast, personal accomplishment showed clear protective effects, yielding positive associations with absorption (r = 0.45; *p* = 0.035), social optimism (r = 0.51; *p* = 0.030), and self-efficacy (r = 0.49; *p* = 0.022; Table A1).

### 3.4. Path Analysis and Moderation Results

As summarized in Table 4, contractual instability showed a large positive effect on burnout (β = 0.45; *p* < 0.001). Burnout, in turn, was strongly and inversely related to humanization (β = −0.52; *p* < 0.001). Humanization displayed a moderate positive association with work engagement (β = 0.38; *p* < 0.001), whereas burnout retained a smaller but significant direct, negative link to engagement (β = −0.24; *p* < 0.001). The direct path from instability to engagement was nonsignificant (β = −0.10; *p* = 0.084), indicating that the impact of contractual instability on engagement operates almost entirely through burnout and reduced humanization.

Bootstrap tests confirmed two robust indirect effects. A two-step pathway, Instability → Burnout → Humanization, produced a standardized indirect coefficient of β = −0.23 (95% CI = −0.31 to −0.15; *p* < 0.001). The full serial chain Instability → Burnout → Humanization → Engagement was likewise significant (β = −0.09; 95% CI = −0.13 to −0.05; *p* < 0.001), accounting for approximately 22% of the total effect.

The moderator analyses appear in Table 4. Age amplified the Instability → Burnout association (interaction β = 0.11; *p* = 0.026): at one standard deviation above the mean age, contractual instability was a strong predictor of burnout (simple slope β = 0.58; *p* < 0.001), whereas the effect was milder but still significant for younger nurses (β = 0.32; *p* = 0.009). Cognitive empathy showed a marginal buffering trend on the Burnout → Engagement path (interaction β = −0.08; *p* = 0.078); the simple-slope contrast did not reach conventional significance. Sex, hospital ownership, and shift pattern failed to moderate any pathway (*p* > 0.10) and are reported in Table A2.

With the aim to check the sensitivity with ordinal instability, we conducted a mediation analysis with the number of temporary contracts recoded into three ordinal terciles (0–1, 2–3, and ≥4). The indirect path, Instability → Emotional Exhaustion → Engagement, remained significant and virtually unchanged (unstandardized B = −0.15; SE = 0.05, 95% BCa CI [−0.27; −0.06], *p* = 0.002). The direct effect of instability on engagement was still nonsignificant (B = −0.08; SE = 0.07; *p* = 0.248). (See Appendix A.3).

## 4. Discussion

To the best of the authors’ knowledge, this is the first study to explore the level of burnout among nursing professionals employed under temporary contracts who are frequently rotated across different hospital units and subjected to irregular and unpredictable shift schedules. The study also examines whether these working conditions affect nurses’ work engagement and their ability to deliver humanized care.

The first objective of this study addressed whether burnout levels vary based on years of professional experience and number of temporary contracts. Our results revealed a large positive correlation year of professional experience and burnout. Although some longitudinal studies have shown a plateau or even decline in burnout with tenure, our findings mirror work conducted in precarious labor contexts, where the long-term uncertainty of successive fixed-term contracts exacerbates emotional fatigue [20].

Emotional exhaustion, the core “health-impairment” component of burnout, displayed the strongest inverse links with both engagement and humanization, replicating Molero Jurado et al.’s (2021) [15] evidence that depleted emotional resources erode empathy, sociability, and self-efficacy. These findings reinforce the idea that cumulative strain over time, particularly under unstable employment conditions, fosters burnout and deteriorates both the quality of care and the professional identity of nurses [32,33].

The second objective of this study was to examine whether the number of contracts signed in the past year has had a negative impact on work engagement. Employment instability also undermined work engagement: the number of contracts in 2023 correlated negatively with overall engagement [ρ = −0.62, *p* < 0.001], and was driven mainly by reduced absorption. This result is consistent with the findings of Cottle-Quinn et al. (2022) [21], who reported that temporary and frequently reassigned nurses often express lower levels of job satisfaction and are more likely to consider leaving the profession. Their study emphasized that nurses under unstable contract arrangements face greater difficulty integrating into work teams and adapting to unit-specific workflows, which diminishes their professional continuity and sense of belonging—two critical elements for maintaining engagement. The authors also highlighted that job retention and engagement improve when nurses perceive their work conditions as stable and when they receive adequate support, recognition, and a healthy work-life balance [34]. Moreover, García-Iglesias et al. (2021) [14] demonstrated that work engagement is not only influenced by job structure, but also by psychosocial risks associated with instability, such as emotional fatigue, loss of autonomy, and a lack of team cohesion. In temporary workers, these factors are exacerbated, especially when rotations and shifts are disorganized or lack orientation support. So, these results suggest that frequent contract renewals and high employment turnover compromise one of the core mechanisms through which engagement is sustained: the ability to form emotional and professional connections with the work environment.

Finally, the third objective explored the relationship between burnout and humanization of care. We observed that burnout was strongly and inversely associated with humanization of care. Sociability and self-efficacy bore the brunt of this relationship, confirming that burnout compromises the relational competencies that are essential for person-centered care [18]. Although the empathy subscale was non-significant, prior systematic reviews suggest that empathy often emerges as a mediator rather than a simple correlate [12,13]. In high-demand environments, such as temporary rotations, empathy functions as a cognitive–affective resource that guides prosocial decision-making and provides a stabilizing force that helps sustain engagement [16,19]. This pattern reinforces the proposed mediation model: employment instability heightens burnout; heightened burnout erodes nurses’ perceptions of humanized care; and this erosion, in turn, dampens work engagement, which is an effect already foreshadowed by studies on empathy-driven prosocial motivation [16,19] and job crafting in nursing [17]. Although a direct correlation between work engagement and humanization was not reported in these results, the parallel inverse associations of both variables with burnout suggest that they may be positively related, as posited in prior frameworks [18]. This opens a compelling avenue for proposing a multiple mediation model whereby employment instability increases burnout, which reduces humanization, which in turn lowers engagement.

### 4.1. Mediation Model Interpretation

**Figure 1:**
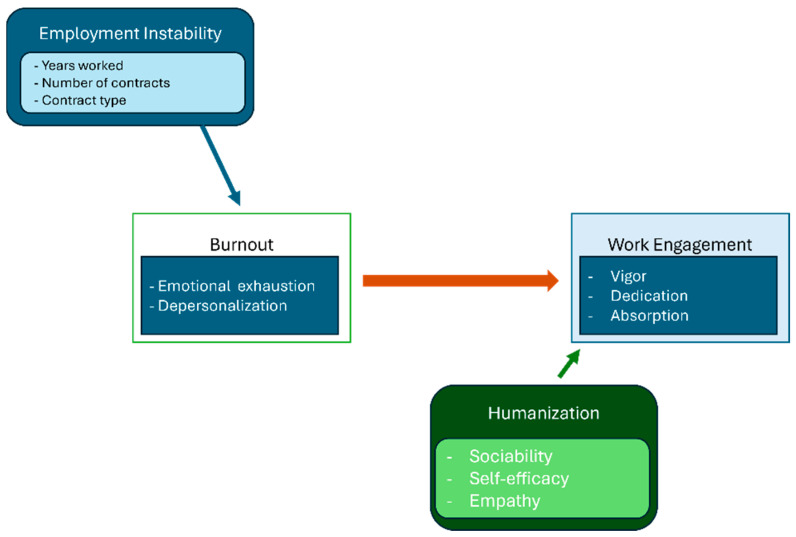
Mediation model.

Our path analysis empirically confirmed the serial mediation originally hypothesized: contractual instability elevates burnout, burnout erodes nurses’ perception of humanized care, and this erosion ultimately lowers work engagement (standardized serial indirect β = −0.09, 95% CI = −0.13 to −0.05, *p* < 0.001). In Job Demands–Resources terms, instability operates as a chronic job demand that triggers a health-impairment process; burnout then depletes humanization, conceived as a personal resource comprising sociability, empathy, self-efficacy, and affectivity in the clinician–patient relationship [15,18], and the loss of that resource undermines the motivational pathway sustaining engagement (Figure 1).

Employment stressors such as frequent temporary contracts, high unit rotation, and the cumulative effect of years in practice predicted higher emotional exhaustion and depersonalization, mirroring prior reports of overload and diminished resilience in precarious nursing contexts [31,35]. Once burnout is established it strongly and inversely affects humanization, with the largest impact on sociability and self-efficacy, which are two competencies that enable meaningful patient interactions. A decline in humanized-care perception, in turn, is associated with lower vigor, dedication, and absorption, which is consistent with earlier evidence that humanization fosters intrinsic motivation, emotional connection, and job meaning in high-demand environments [16,19].

The empirically supported pathway identifies two leverage points: (i) Stabilizing staffing arrangements, lengthening contract duration, and limiting non-essential rotations can reduce burnout at its origin. (ii) Strengthening humanization-related skills (empathy, sociability, and self-efficacy) may buffer the downstream impact on engagement even when some degree of contractual precarity persists. These measures carry operational benefits: lower burnout curbs absenteeism and turnover, which currently cost hospitals an estimated USD 37,000–58,000 per nurse per year and up to USD 31,000 per facility per day when left unaddressed [36,37,38]. Burnout is also linked to medical errors and longer lengths of stay, further eroding financial margins [38].

Workforce stabilization yields environmental dividends as well. High turnover multiplies orientation sessions, duplicates training materials, and increases single-use consumption during onboarding; cohesive, long-tenured teams can streamline processes and cut redundant procurement, thereby advancing environmental stewardship [39].

### 4.2. Limitations and Future Research Implications

This study has several limitations. Foremost, its cross-sectional nature precludes causal inference, and self-report measures may inflate associations via a common-method bias. Our sample, Spanish hospital nurses on temporary contracts, restricts generalizability, and non-responders could differ systematically from participants. Objective indicators [e.g., contract records and patient outcomes] and potential buffers such as organizational support or emotional intelligence were not assessed. Although the nursing workforce in Spain is predominantly female, the over-representation of women in our sample may restrict the generalizability of the findings to male nurses. Future studies should consider stratified sampling or sex-specific sensitivity analyses to ensure robustness across genders. Furthermore, because participation was voluntary and the survey was administered online, we cannot exclude the possibility that individuals with greater digital access or stronger opinions were more likely to respond. In addition, because only fixed-term nurses were surveyed, comparisons with colleagues on permanent contracts could not be made; future work should incorporate a control group to gauge the incremental burden of contractual precarity. Finally, we captured instability only through the number of temporary contracts and did not include a comparison group of permanently employed nurses; thus, generalization beyond fixed-term personnel should be made with caution.

In relation with future research, it could be necessary follow nurses longitudinally, combine survey data with administrative and patient metrics, and test the proposed serial mediation using time-lagged structural-equation models. Multi-level designs can disentangle individual and unit influences, while intervention trials, such as extending contract length or providing empathy or resilience training, are needed to verify whether stabilizing employment or strengthening humanization resources truly reduces burnout and boosts engagement.

## 5. Conclusions

This investigation describes the psychosocial and organizational correlates of contractual precariousness among Spanish hospital nurses on fixed-term agreements. Our cross-sectional data show that employment instability, operationalized as the cumulative number of temporary contracts and unit reassignments, was associated **with** higher burnout, lower work engagement, and weaker perceptions of humanized care. Emotional exhaustion emerged as the principal burnout component, exerting concurrent negative effects on engagement and the relational competencies underpinning person-centered practice.

The pattern of associations is compatible with a serial mediation framework in which greater contractual instability may be linked to higher burnout, which may co-occur with lower humanization and, consequently, reduced engagement. Longitudinal evidence is required to confirm this temporal sequence.

From an organizational perspective, these findings suggest that interventions such as extending contract durations, limiting non-essential unit rotations, and offering resilience programs that foster humanization-related skills could be beneficial; nonetheless, experimental studies are needed to test their impact. Such measures are expected to moderate emotional exhaustion, preserve humanizing competencies, and ultimately enhance patient care quality. Any application of these recommendations outside the Spanish hospital context should consider local labor regulations, staffing models, and cultural factors.

Given the cross-sectional design, causal inference is precluded, so future studies should employ longitudinal or quasi-experimental designs to verify the proposed mediation pathway, incorporate objective indicators [e.g., administrative contract data and patient-reported outcomes], and assess moderating variables such as perceived organizational support and emotional intelligence. These endeavors will refine the evidence base for optimizing working conditions and safeguarding the psychological well-being and professional efficacy of an increasingly contingent nursing workforce.

## Figures and Tables

**Table 1:** Sociodemographic characteristics of temporary-contract nurses.

Variable	Category	n (%)
Gender	Male	108 (27.0%)
	Female	292 (73.0%)
Marital Status	Single	140 (35.0%)
	Divorced	108 (27.0%)
	Married	152 (38.0%)
Hospital Type	Public	232 (58.0%)
	Private	168 (42.0%)
Work Shift	Morning	127 (31.8%)
	Afternoon	12 (3.0%)
	Rotating	261 (65.3%)

**Table 2:** Descriptive statistics for occupational variables and psychometric scales.

Variable		Mean (SD)	CI 95%
Age (years)		38.53 (9.61)	(37.59–39.48)
Experience (years)		14.55 (7.74)	(12.07–17.03)
Number of contracts		2.67 (3.068)	(2.37–2.97)
Burnout (MBI)		2.99 (0.44)	(2.94–3.03)
Engagement (UWES)	Total	3.00 (0.47)	(2.95–3.04)
	Vigor	3.13 (0.42)	(3.09–3.17)
	Dedication	2.95 (0.51)	(2.9–3.0)
	Absorption	2.97 (0.49)	(2.93–3.02)
Humanization (HUMAS)	Total	2.49 (0.39)	
	Optimism	2.67 (0.42)	(2.63–2.71)
	Sociability	2.61 (0.37)	(2.57–2.64)
	Emotional Understanding	2.45 (0.35)	(2.41–2.48)
	Self-Efficacy	2.53 (0.32)	(2.5–2.56)
	Affection	2.3 (0.42)	(2.26–2.34)

Note: MBI = Maslach Burnout Inventory; UWES = Utrecht Work Engagement Scale; and HUMAS = Health Professionals’ Humanization Scale.

**Table 3:** Association results between burnout, work engagement, humanization, and sociodemographic variables.

		Age (r; *p*)	Experience (Years) (r; *p*)	Number of Contracts (r; *p*)
Burnout		r = 0.001; *p* = 0.984	0.74; *p* = 0.04	0.42; *p* = 0.039
Engagement (UWES)	Total	0.073; *p* = 0.145	−0.025; *p* = 0.877	−0.62; *p* = 0.018
	Vigor	0.045; *p* = 0.301	−0.015; *p* = 0.841	−0.49; *p* = 0.025
	Dedication	0.036; *p* = 0.408	−0.019; *p* = 0.769	−0.41; *p* = 0.033
	Absorption	0.051; *p* = 0.287	−0.010; *p* = 0.902	−0.39; *p* = 0.041
Humanization (HUMAS)	Total	0.045; *p* = 0.372	−0.244; *p* = 0.129	−0.043; *p* = 0.392
	Optimism	0.032; *p* = 0.511	−0.12; *p* = 0.241	−0.08; *p* = 0.343
	Sociability	0.021; *p* = 0.672	−0.26; *p* = 0.101	−0.02; *p* = 0.776
	Emotional Understanding	0.037; *p* = 0.441	−0.17; *p* = 0.193	−0.05; *p* = 0.509
	Self-Efficacy	0.039; *p* = 0.393	−0.20; *p* = 0.144	−0.10; *p* = 0.321
	Affection	0.015; *p* = 0.782	−0.18; *p* = 0.175	−0.06; *p* = 0.462

**Table 4:** Path analysis.

**Block A. Primary Paths**	**Std. β**	**SE**	***p***	**95% CI**		
Burnout ← Temporary contract count	0.45	0.05	<0.001	(0.36; 0.55)		
Humanization ← Burnout	−0.52	0.06	<0.001	(−0.63; −0.40)		
Engagement ← Humanization	0.38	0.06	<0.001	(0.26; 0.50)		
Engagement ← Burnout	−0.24	0.05	<0.001	(−0.34; −0.14)		
Engagement ← Temporary contract count (direct)	−0.10	0.06	0.084	(−0.22; 0.01)		
**Block B. Moderators**	**Moderated Path**	**Interaction β**	***p***	**95% CI**	**Simple Slope (+1 SD) †**	**Simple Slope (−1 SD) †**
Age (centered, years)	Instability → Burnout	0.11	0.026	(0.01; 0.21)	β = 0.58 ***	β = 0.32 **
Cognitive Empathy (HUMAS)	Burnout → Engagement	−0.08	0.078 ‡	(−0.17; 0.01)	β = −0.32	β = −0.15

Note: † Simple slopes computed at ±1 SD of the moderator. ‡ Trend-level effect (0.05 < *p* < 0.10). Std. β = standardized coefficient; CI = confidence interval derived from 5000 bootstrap resamples. ** *p* < 0.01 and *** *p* < 0.001.

## Data Availability

The dataset analyzed in the current study is not publicly available due to national data regulations and for ethical reasons, including that we do not have the explicit written consent of the study volunteers to make their deidentified data available at the end of the study. However, datasets can be requested by sending a letter to the corresponding author.

## Public Involvement Statement

Healthcare professionals—nurses—were involved in this study about health and social care research. No patients, consumers, or informal carers were involved in this study.

## Guidelines and Standards Statement

This manuscript was drafted against the STROBE guidelines for observational studies.

## Appendix A

### Appendix A.1

**Table A1:** Correlations of burnout dimensions with work engagement and humanization subscales.

		UWES				HUMAS					
		Total	VIG	DED	ABS	Total	OPT	SOC	EMP	SE	AFF
**MBI**	Total	r = −0.54; *p* = 0.028	r = −0.5; *p* = 0.03	r = −0.52; *p* = 0.025	r = −0.3; *p* = 0.034	r = −0.61; *p* = 0.032	r = −0.11; *p* = 0.029	r = −0.62; *p* = 0.019	r = −0.03; *p* = 0.621	r = −0.62; *p*= 0.002	r = −0.027; *p* = 0.592
	EE	r = −0.6; *p* = 0.015	r = −0.58; *p* = 0.02	r = −0.55; *p* = 0.022	r = −0.32; *p* = 0.04	r = −0.64; *p* = 0.021	r = −0.14; *p* = 0.041	r = −0.580; *p* = 0.025	r = −0.04; *p* = 0.598	r = −0.66; *p* = 0.001	r = −0.04; *p* = 0.56
	DP	r = −0.45; *p* = 0.04	r = −0.42; *p* = 0.054	r = −0.43; *p* = 0.046	r = −0.28; *p* = 0.037	r = −0.5; *p* = 0.037	r = −0.08; *p* = 0.072	r = −0.6; *p* = 0.033	r = −0.02; *p* = 0.701	r = −0.51; *p* = 0.018	r = −0.03; *p* = 0.61
	PA	r = 0.4; *p* = 0.045	r = 0.38; *p* = 0.048	r = 0.41; *p* = 0.042	r = 0.45; *p* = 0.035	r = 0.45; *p* = 0.035	r = 0.11; *p* = 0.056	r = 0.51; *p* = 0.03	r = 0.5; *p* = 0.04	r = 0.49; *p* = 0.022	r = 0.06; *p* = 0.58

Note: MBI: Burnout; EE = Emotional Exhaustion; DP = Depersonalization; and PA = Reduced Personal Accomplishment [reverse-scored]. UWES: VIG = Vigor, DED = Dedication, and ABS = Absorption. HUMAS: OPT = Optimism, SOC = Sociability, EMP = Empathy, SE = Self-Efficacy, and AFF = Affection.

### Appendix A.2

**Table A2:** Non-significant moderators of the temporary contract → Burnout path.

Moderator (Coding)	Moderated Path	Interaction β (std.)	SE	Z (*p*)	95% CI (Bootstrap)
Sex (0 = male, 1 = female)	Temporary contract → Burnout	0.04	0.04	1.18 (0.24)	(−0.03; 0.11)
Hospital type (0 = private, 1 = public)	Temporary contract → Burnout	0.03	0.03	1.01 (0.31)	(−0.03; 0.08)
Shift pattern (0 = fixed, 1 = rotating)	Temporary contract → Burnout	0.05	0.04	1.32 (0.19)	(−0.02;0.12)

Note: Std. β = standardized coefficient. None of the interactions reached statistical significance (*p* > 0.10). No additional simple-slope analyses were performed.

### Appendix A.3

**Table A3:** Sensitivity analysis with ordinal terciles of temporary-contract count.

Path	Measure	Unstandardized B	SE	95% BCa CI	*p*
Temporary contracts count (tercile) → Burnout (EE)	a_1_	0.47	0.14	(0.20; 0.77)	0.001
Burnout (EE) → Engagement (UWES total)	b_1_	−0.31	0.09	(−0.48; −0.149)	0.001
Indirect effect (a_1_ × b_1_)	—	−0.15	0.05	(−0.27; −0.06)	0.002
Direct effect Temporary contracts count → Engagement	c′	−0.08	0.07	(−0.21; 0.05)	0.248

Note: Temporary-contract count was recoded into three ordinal categories: low (0–1 contract, reference = 0), medium (2–3), and high (≥4). Terciles were entered as two dummy variables; table values correspond to the linear contrast (0, 1, and 2).
